# [Corrigendum] Mechanistic role of the ILK-S100A4 axis in modulating invasion and EMT in salivary adenoid cystic carcinoma

**DOI:** 10.3892/ol.2026.15489

**Published:** 2026-02-12

**Authors:** Yang Yang, Jia-Xin Luo, Yuan-Yang Li, Ke-Hong Guo, Lin Ye, Dan Zhao

Oncol Lett 30: 597, 2025; DOI: 10.3892/ol.2025.15343

Subsequently to the publication of the above paper, an interested reader drew to the Editor's attention that a pair of data panels shown for the scratch-wound assay experiments in Figs. 1 and 7 were apparently overlapping; in addition, another pair of data panels within Fig. 7 itself were also found to be overlapping.

Upon examining their original raw data (which were also presented to the Editorial Office for our inspection), the authors realized that several inadvertent errors had been made in the presentation of Figs. 2 and 7 in the published paper. Specifically, the image for the 24 h, SACC-LM Control (CON) group in Fig. 7 was inadvertently duplicated and used for the 24 h, negative control (NC-S100A4) group of the same cell line. Secondly, the image intended for the 24 h, SACC-LM sh-ILK group in Fig. 7 had mistakenly been replaced by an image from a separate sh-ILK experiment performed with the SACC-83 cells. Thirdly, the western blot data depicting ILK and Snail expression in the SACC-83 cell line in Fig. 2 were inadvertently re-used to represent different experimental endpoints in Figs. 3 and 6.

The revised versions of Figs. 2 and 7, featuring the correct data for the ILK and Snail blots for the SACC-83 cell line in Fig. 2, and the correct data for the 24 h, NC-S100A4 and 24 h, sh-ILK experiments for the SACC-LM cell line in Fig. 7, are shown on the next two pages. The authors regret these errors were made in compiling these figures, which resulted from administrative oversights during the image selection and integration process, although note that these errors did not grossly affect either the results or the conclusions reported in this study. The authors are grateful to the editor of *Oncology Letters* for allowing them the opportunity to publish a Corrigendum; furthermore, they apologize to the readership for any inconvenience caused.

## Figures and Tables

**Figure 2.**
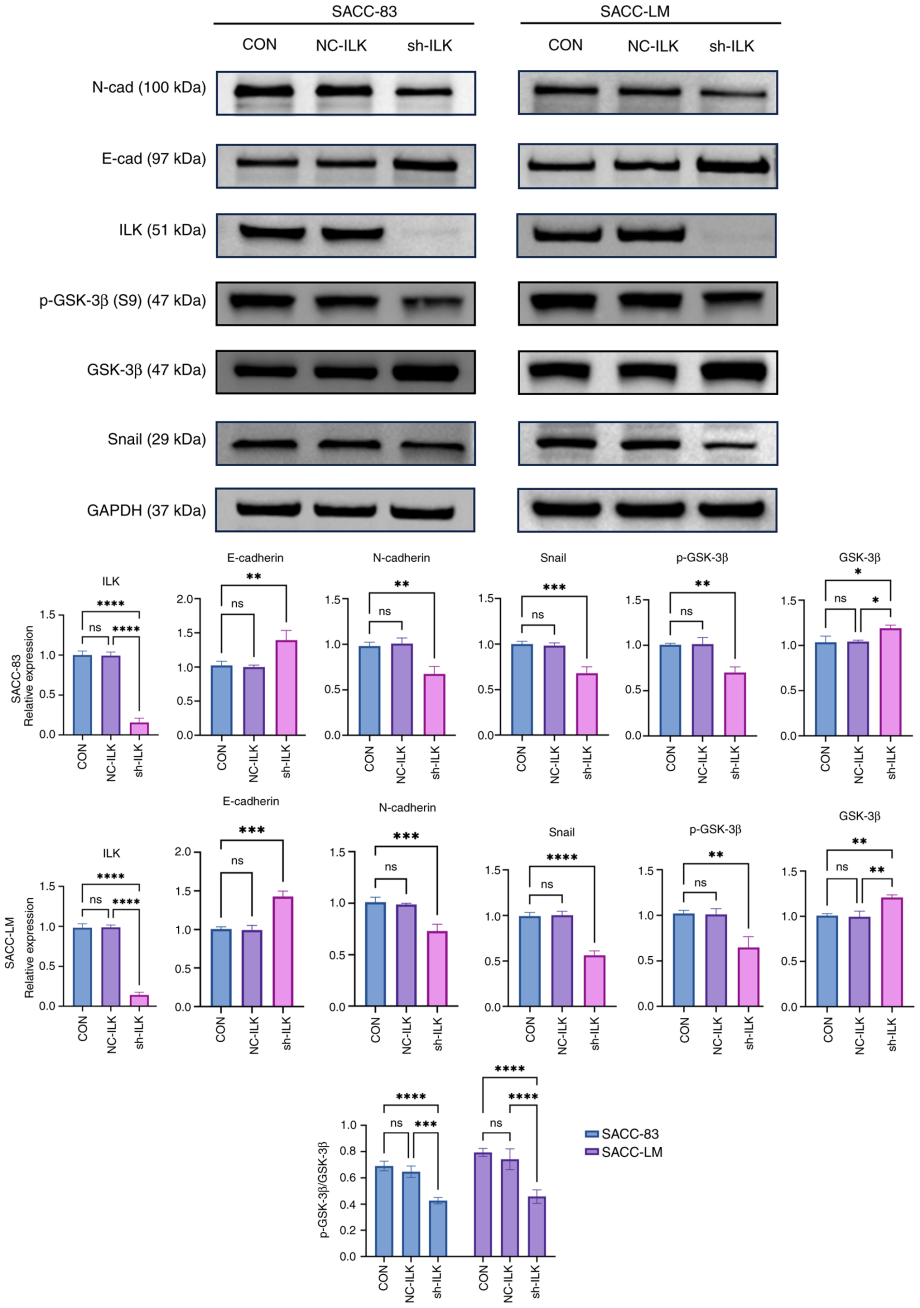
Western blot analysis of the effects of ILK knockdown on EMT markers and signaling. Results are shown as the mean ± SD of three independent assays. *P<0.05, **P<0.01, ***P<0.001, ****P<0.0001 vs. control group. ns, not significant.

**Figure 7.**
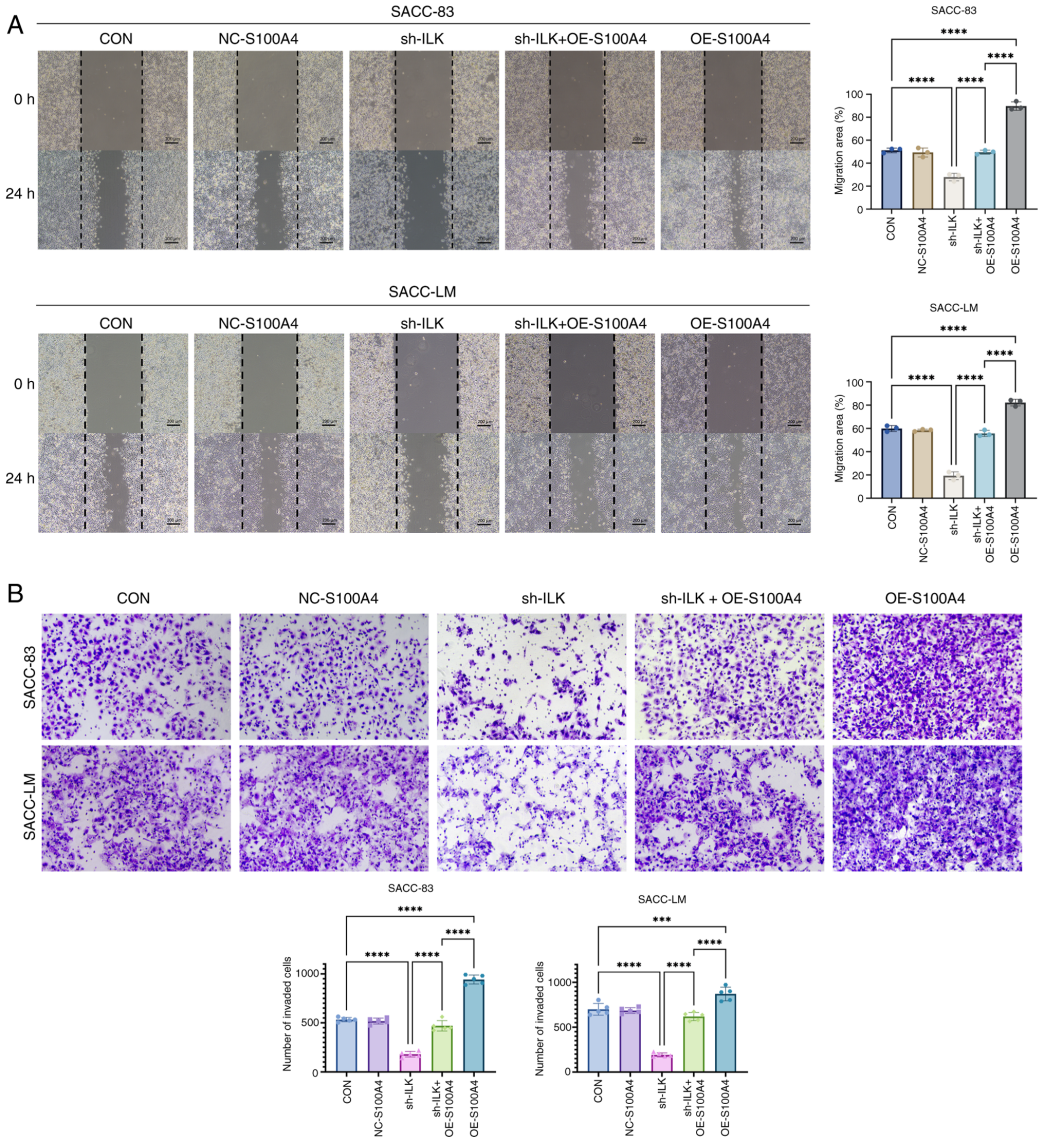
S100A4 OE rescues the inhibitory effects of ILK knockdown on the migration and invasion of adenoid cystic carcinoma. (A) Wound healing assays (10X magnification) demonstrate that S100A4 OE restores migratory ability of SACC cells, with sh + OE cells showing closure rates comparable with NC. (B) Transwell assays revealed restoration of invasive capacity in sh + OE cells suggests a reversal of ILK knockdown-induced inhibition. ***P<0.001, ****P<0.0001. ns, not significant; OE, overexpression; ILK, integrin-linked kinase; SACC, salivary adenoid cystic carcinoma; sh, short hairpin; NC, negative control; CON, blank control.

